# Enteroendocrine cells and gut hormones as potential targets in the crossroad of the gut-kidney axis communication

**DOI:** 10.3389/fphar.2023.1248757

**Published:** 2023-10-19

**Authors:** José Arimatéa de Oliveira Nery Neto, Victor Yuji Yariwake, Niels Olsen Saraiva Câmara, Vinicius Andrade-Oliveira

**Affiliations:** ^1^ Bernardo’s Lab, Center for Natural and Human Sciences, Federal University of ABC, Santo André, Brazil; ^2^ Laboratory of Transplantation Immunobiology, Department of Immunology, Institute of Biomedical Sciences, University of São Paulo, São Paulo, Brazil

**Keywords:** kidney diseases, enteroendocrine cells, renal inflammation, gut-kidney axis, remote signaling

## Abstract

Recent studies suggest that disruptions in intestinal homeostasis, such as changes in gut microbiota composition, infection, and inflammatory-related gut diseases, can be associated with kidney diseases. For instance, genomic investigations highlight how susceptibility genes linked to IgA nephropathy are also correlated with the risk of inflammatory bowel disease. Conversely, investigations demonstrate that the use of short-chain fatty acids, produced through fermentation by intestinal bacteria, protects kidney function in models of acute and chronic kidney diseases. Thus, the dialogue between the gut and kidney seems to be crucial in maintaining their proper function, although the factors governing this crosstalk are still emerging as the field evolves. In recent years, a series of studies have highlighted the significance of enteroendocrine cells (EECs) which are part of the secretory lineage of the gut epithelial cells, as important components in gut-kidney crosstalk. EECs are distributed throughout the epithelial layer and release more than 20 hormones in response to microenvironment stimuli. Interestingly, some of these hormones and/or their pathways such as Glucagon-Like Peptide 1 (GLP-1), GLP-2, gastrin, and somatostatin have been shown to exert renoprotective effects. Therefore, the present review explores the role of EECs and their hormones as regulators of gut-kidney crosstalk and their potential impact on kidney diseases. This comprehensive exploration underscores the substantial contribution of EEC hormones in mediating gut-kidney communication and their promising potential for the treatment of kidney diseases.

## 1 Introduction

The kidneys are essential organs responsible for various important biological processes. Some of the main functions of kidneys include blood filtration, which occurs in the nephrons, maintaining nutrients and ions homeostasis, producing substances associated with the renin-angiotensin system for regulating the cardiovascular system, and participating in the blood pH regulation, as well as producing vitamin D and other hormones such as erythropoietin and thrombopoietin (Kellum et al., 2021). Due to the central role of kidneys in the maintenance of organismal homeostasis, the study of diseases that affect the kidneys is crucial. Acute kidney injury (AKI) refers to abrupt decline in the kidney function, often triggered by cardiovascular impairments, infections, or sepsis, leading to a low glomerular filtration rate and kidney failure. Chronic kidney diseases (CKDs) manifest as a gradual and continuous degeneration of the kidneys, which can occur by a variety of causes, usually associated with hypertension, diabetes, and immunological dysfunctions (Imig and Ryan, 2013). Therefore, understanding the factors associated with the development or aggravation of nephropathies is essential for the constitution of prevention methods, treatment improvements, and patient management.

**FIGURE 1:**
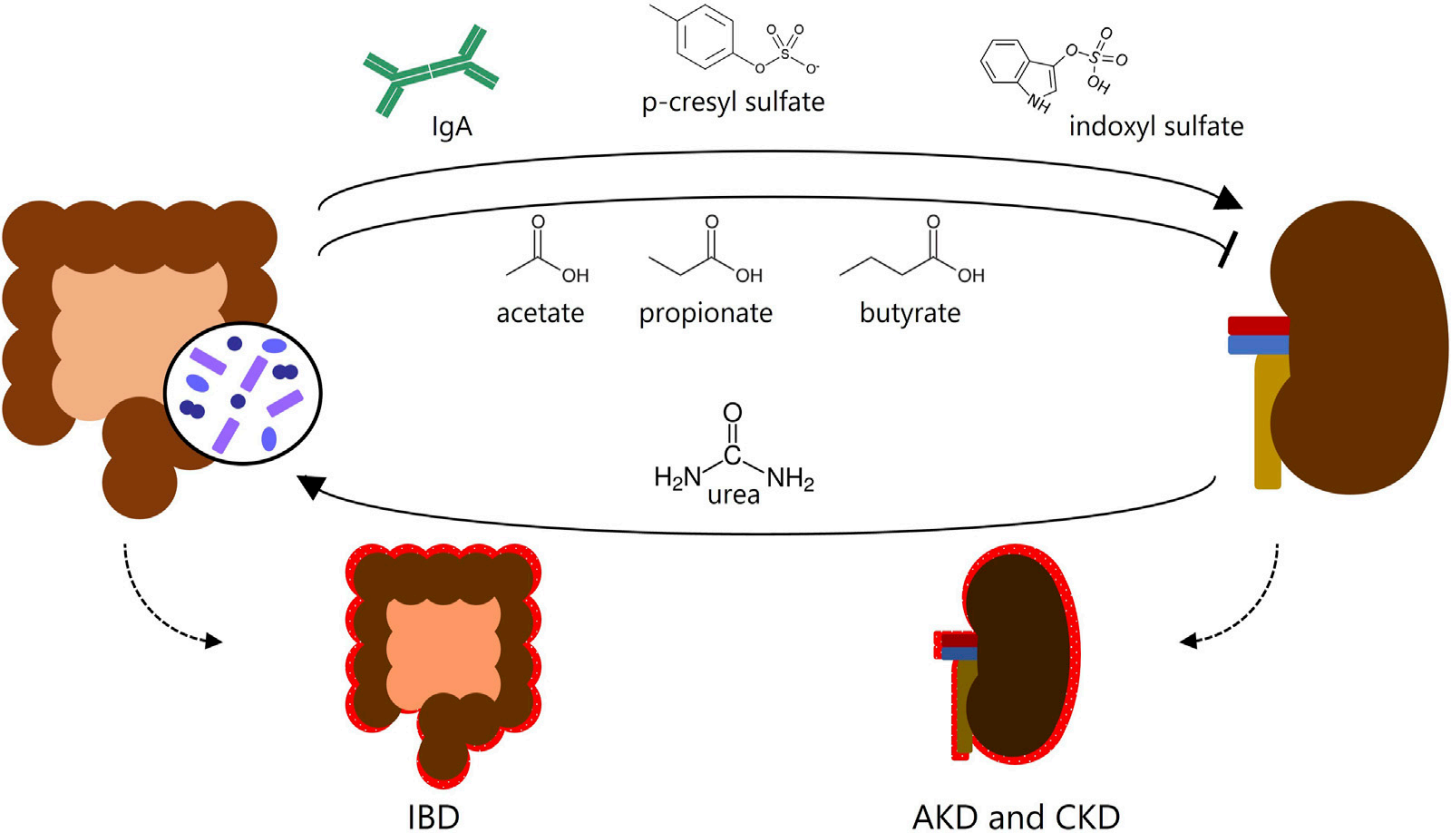
Gut-kidney crosstalk. Known metabolites produced in the intestine affect the kidneys and may cause or prevent kidney inflammation, and *vice versa*. IBD, inflammatory bowel disease; AKD, acute kidney disease; CKD, chronic kidney disease.

One factor rarely explored, despite the increasing evidence, is the association between inflammatory bowel diseases (IBD) and kidney diseases. Recent studies suggest that the inflammatory processes in IBD may impair renal function (Ambruzs and Larsen, 2018; Chang et al., 2019; Vajravelu et al., 2020; Li et al., 2022). Uncovered mechanisms involved in this dialogue between the intestine and kidneys account for enhancing kidney inflammation, such as proteins and metabolites produced in the intestine that may cause kidney inflammation (e.g., immunoglobulin A (IgA) deposition, p-cresyl sulfate, and indoxyl sulfate) (Han et al., 2016; Lin et al., 2019; Cheng et al., 2020) whereas others prevent kidney lesions (e.g., short-chain fatty acids (SCFAs)) (Andrade-Oliveira et al., 2015) (Figure 1). The complexity of both organs suggests that more mechanisms take place in this intricate relationship.

Intestinal epithelial cells (IECs), for instance, mediate the crosstalk between the lumen content and the mucosal cells and elicit physical and chemical responses that can influence the body systemically (Soderholm and Pedicord, 2019). Among these cells, enteroendocrine cells (EECs) seem to be particularly relevant due to the production of EEC hormones that are released in the bloodstream reaching distant organs. Interestingly, EEC hormone receptors are expressed in several cells and tissues throughout the body, raising the possibility of other organs being modulated by EEC hormones. For example, renal cells have receptors for some hormones produced by EECs (Zietek and Rath, 2016). Controversially, the role of EECs and the plausible mechanisms of crosstalk between EECs and kidneys in the context of nephropathies have not been explored so far.

Therefore, this review explores and analyzes data associated with the hypothesis of crosstalk between the intestine and kidneys mediated by EEC hormones in the context of renal diseases. For this purpose, we integrated studies from PubMed and randomized clinical trials and performed *in silico* analyses of transcriptomes (microarray and RNAseq) obtained from humans and mice.

## 2 Gut-kidney crosstalk

The interest in the crosstalk between the intestine and kidneys has been increasing in the last few years. A series of studies demonstrate a direct relationship between these organs regarding health and disease states. For instance, IgA secretion in the intestine influences the balance between commensal and pathogenic bacteria. This immunoglobulin is involved in the pathogenesis of IgA nephropathy (IgAN), which is the most common primary glomerulonephropathy worldwide (Felizardo et al., 2016). The pathophysiology of IgAN involves the deposition of abnormal IgA within the glomeruli. These antibodies, originating from IgA-producing intestinal plasma cells that, somehow, are not directed to the mucosal surfaces to neutralize commensal microbes. Instead, they are released into the bloodstream and deposited in the glomeruli, triggering and sustaining local inflammatory reactions and causing a decline in kidney function (Knoppova et al., 2021). Genomic investigations demonstrate that certain IgAN susceptibility genes are also associated with the risk of developing IBD (Han et al., 2016). Furthermore, the efficacy of treating IgAN has been demonstrated using drugs intended for the treatment of intestinal inflammation (Sanchez-Russo et al., 2022).

Diabetic nephropathy (DN) is another disease in which the connection between the gut and kidneys becomes more evident. In DN, the kidneys are adversely affected by diabetes, a condition characterized by hyperglycemia and heightened gut permeability (Sabatino et al., 2017). Consequently, the heightened levels of lipopolysaccharide (LPS) resulting from compromised intestinal integrity are linked to CKD conditions and progress towards end-stage renal diseases (ESRD) (Monteiro and Berthelot, 2021). One conceivable mechanism involves the recognition of LPS by immune cells residing in the kidneys, thus triggering processes of kidney inflammation (Huang et al., 2017; Foresto-Neto et al., 2021). Beyond the leaky gut mechanism, the dysbiosis caused by the consumption of unbalanced diets can also contribute to the development of kidney dysfunction. The excessive intake of food can be metabolized by the microbiota, enhancing the production of trimethylamine, which is converted to trimethylamine N-oxide (TMAO) by liver cells. This compound is associated with kidney damage and the progression of CKD (Stavropoulou et al., 2020; Foresto-Neto et al., 2021). Together, these findings suggest the existence of interesting mechanisms involving the role of microbiota in the gut-kidney crosstalk.

An important factor related to intestinal disturbance of the microbiota, is the production of toxic metabolites known as uremic toxins, such as p-cresyl sulfate and indoxyl sulfate (Di Iorio et al., 2017). These toxins have been shown to contribute to the progression of kidney disease in both humans and mice (Crespo-Salgado et al., 2016; Yoshifuji et al., 2016). P-cresyl sulfate is a byproduct of the metabolism of the amino acids tyrosine and phenylalanine by anaerobic bacteria in the intestine. It undergoes the action of cytosolic sulfotransferases along the intestinal mucosa, which can convert p-cresol into p-cresyl sulfate (Liabeuf et al., 2010). An increase in its free form in the blood is associated with vascular calcification, arterial stiffness, and increased mortality risk in patients with CKD and those undergoing hemodialysis (Lin et al., 2019). This increase is likely due to the enhanced production of reactive oxygen species (ROS) and pro-oxidant effects on human tubular epithelial cells, along with the increased activity of NADPH oxidase and levels of inflammatory cytokines associated with renal fibrosis (Watanabe et al., 2013). Another important amino acid metabolized in the colon is tryptophan, which is converted to indole by the microbiota and enters the systemic circulation. It is subsequently metabolized by the liver to form indoxyl sulfate (Leong and Sirich, 2016). Indoxyl sulfate is filtered in the kidneys and can induce tubular cell death, increase oxidative stress, and stimulate renal fibrosis through the overproduction of transforming growth factor beta (TGF-β) (Cheng et al., 2020). Oral administration of AST-12, an indoxyl sulfate binder, prevented glomerular sclerosis in early-stage renal failure in rats that underwent subtotal nephrectomy (Kobayashi et al., 2002).

Progressive renal insufficiency, on the other hand, results in higher concentrations of urea in the blood, which can reach the intestine and be degraded by bacterial ureases, leading to hydrolysis into ammonia and easily converted into ammonium hydroxide. This culminates in an increase in faecal pH followed by alteration of intestinal cell junctions, irritation of the mucosa, and enterocolitis (Vaziri, 2012; Di Iorio et al., 2017). Thus, uremia contributes to increased intestinal permeability, which can subsequently result in the translocation of bacteria and endotoxins across the intestinal wall (Ramezani and Raj, 2014). In light of this, elevated levels of bacterial products in circulation have been observed in patients with CKD compared to healthy individuals (Yang et al., 2018). Therefore, intestinal changes may be the key factor contributing to renal inflammation in patients with advanced CKD (Meijer et al., 2018). In fact, after a median follow-up of 4.9 years, patients with IBD had a significantly higher risk of developing ESRD compared to controls (adjusted hazard ratio = 3.03; 95% confidence interval: 1.77–5.20; *p* < 0.001) (Park et al., 2018).

Several studies have demonstrated that SCFAs, which are produced from the fermentation of complex carbohydrates by the intestinal microbiota, have beneficial effects on renal function. For example, acetate and butyrate have been observed as protectors in models of AKI and CKD, respectively (Andrade-Oliveira et al., 2015; Felizardo et al., 2019). Additionally, SCFAs are used as an energy source for colonocytes, contributing to the maintenance of the intestinal epithelium (Li et al., 2017). This preservation of the intestinal epithelial barrier has anti-inflammatory effects, such as inhibiting activation of the nuclear factor kappa B (NF-κB) transcription factor, and production of interleukin-8 (IL-8) and IL-18 (Huang et al., 2017). Moreover, SCFAs modulate the secretion of EEC hormones, such as peptide YY (PYY) (Freeland and Wolever, 2010), glucagon-like peptide-1 (GLP-1) (Tolhurst et al., 2012), and leptin. Which may partially explain the benefits of SCFAs on energy metabolism (Li et al., 2017). These hormones can be transported by the bloodstream to distant organs such as the kidneys and exert paracrine effects (Latorre et al., 2016).

Thus, the microbiota seems to have an important role in the gut-kidney axis. Sustaining gut homeostasis fosters the continuous absorption of beneficial substances from a “healthy microbiota”, such as SCFAs, which shield kidney function against inflammatory processes (Zhu et al., 2021). On the other hand, gut dysbiosis, abnormal IgA deposition, and harmful metabolites generated by the microbiota (e.g., TMAO, p-cresyl sulfate, and indoxyl sulfate) can exacerbate gut inflammation and kidney lesions, thereby deteriorating the prognosis of CKD and IBD (Cao et al., 2022) (Figure 1). Therefore, the intricate interplay between the microbiota and IECs assumes a critical role. This interplay ensures the proper functioning of the intestine as both an absorptive epithelium and an effective barrier. Additionally, the intestine serves as a proficient communicator with other organs, involving endocrine function through the hormones produced by EECs within the intestinal epithelia.

## 3 EEC biology and communication with the kidneys

The intestinal barrier is composed of the microbiota, mucus, IECs, and the subjacent mucosal immune system (Odenwald and Turner, 2017; Gao et al., 2019). IECs are arranged in a single layer on the luminal surface of the intestinal epithelium and are responsible for maintaining a physical and chemical barrier (Chelakkot et al., 2018). These cells are derived from stem cells that have G protein-coupled receptors (GPCRs) (Scopelliti et al., 2014), such as LGR5 (commonly used as intestinal stem cell (ISC) marker), and that are localized in the depth of intestinal crypts (Guo et al., 2022). Under specific stimuli, ISC differentiates into absorptive enterocytes (Kong et al., 2018), tuft cells (Hendel et al., 2022), Paneth cells that produce antimicrobial peptides like lysozyme (Vaishnava et al., 2011), goblet cells that secrete mucus (Odenwald and Turner, 2017) or EECs. Although EECs constitute only 1% of IECs (Moran-Ramos et al., 2012), they collectively form the largest endocrine organ in the body and are characterized by the production and secretion of a diverse array of hormones (Engelstoft et al., 2013). There are at least 15 subtypes of EECs responsible for the secretion of more than 20 hormones, including ghrelin and nesfatin-1 (A cells), somatostatin (D cells), gastrin (G cells), cholecystokinin (I cells), glucose-dependent insulin-releasing polypeptide and xenin (K cells), GLP-1, GLP-2, glicentin, oxyntomodulin, PYY and insulin-like peptide 5 (L cells), motilin (M cells), neurotensin (N cells), leptin (P cells), secretin (S cells), serotonin and tachykinin (enterochromaffin cells), and histamine (enterochromaffin-like cells) (Sei et al., 2011; Guo et al., 2022). Initially, these cells were classified based on the hormones produced. However, with advances in the sequencing techniques and analyses at the single-cell level it was possible to decipher that some of them are capable of producing and secreting more than one kind of hormone (Moran-Ramos et al., 2012; Haber et al., 2017; Beumer et al., 2018).

**FIGURE 2:**
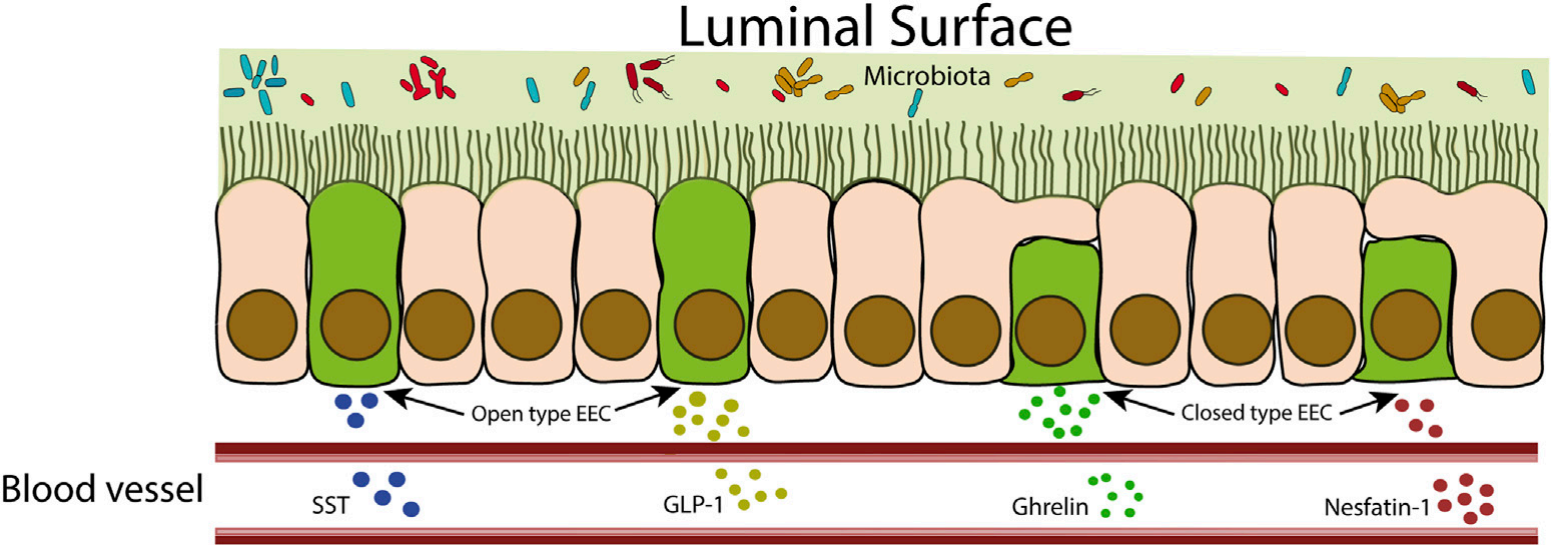
Schematic representation of closed-type and open-type EECs. Open-type EECs are in direct contact with the intestinal lumen. These cells respond to intestinal contents by releasing hormones that can act locally or reach the bloodstream to act at distant sites. Closed cells, as they are not in direct contact with the intestinal lumen, receive stimuli indirectly from neuronal and hormonal signals.

EECs can be classified into “open type” or “closed type” based on their morphology. Open-type EECs have microvilli that protrude and come into direct contact with the intestinal lumen, while closed-type EECs lack microvilli and do not have direct contact with the luminal contents (Figure 2) (Latorre et al., 2016). Open-type EECs express apical receptors designed to perceive signals from the gut microbiota and microbial products. This category of receptors encompasses toll-like receptors (TLR), GPCRs, and taste receptors. TLRs recognize conserved structures from gut bacteria, such as peptidoglycans, flagellins and LPS. Microbiota fermentation products like SCFAs and long-chain fatty acids bind to GPCRs. Furthermore, taste receptors have the ability to detect nutrients and other metabolites produced by the microbiota. This sensing mechanism triggers intracellular signaling within EEC, ultimately culminating in the production and secretion of hormones (Yu et al., 2020). These hormonal responses exert subsequent influences on various aspects, including gastrointestinal motility, mucosal immunology, and even kidney functions. These impacts will be addressed for each EEC hormone in the subsequent sections.

Recently, there is an increasing interest in the hormones produced by EECs (Table 1), due to their action on extraintestinal organs and their potential therapeutic applications (Gribble and Reimann, 2016). Indeed, EECs are distributed throughout the entire gastrointestinal (GI) tract and play a crucial role in the process of secretion, GI motility, regulation of food intake, postprandial glucose levels, and metabolism (Latorre et al., 2016). It has been demonstrated that some of the hormones secreted by EECs, such as the incretins GLP-1 and GLP-2, have renoprotection effects (Sharkovska et al., 2014). GLP-2 also can reduce intestinal permeability and systemic inflammatory phenotype (Cani et al., 2009). As we mentioned before, the existence of communication between the intestine and the kidneys may be supported by the expression of hormone receptors in the renal tissue, for the EEC hormones, suggesting that they can exert some effects in the kidney (Seino and Yabe, 2013; Boer et al., 2022). In this review, we discuss the findings considering each EEC hormone and its receptors in different kidney diseases. To further explore and get more insights into the intricate relationship between the intestine and the kidneys, we also included in this review the evaluation of the expression of receptors for EEC hormones in kidneys samples biopsies from different AKD using publicly available data sets from Microarray and RNA-seq at GEO DataSets (https://www.ncbi.nlm.nih.gov/gds). Accession numbers to the GEO datasets analyzed in this study are: GSE32591; GSE38117; GSE39548; GSE197699; GSE87212; GSE42303; GSE106993; GSE60088; GSE86425; GSE32583; GSE30122; GSE181380; GSE38117; GSE116626; GSE116626; GSE93798; GSE37133; GSE181380. The differentially expressed genes (DEGs) were analyzed via GEO2R (https://www.ncbi.nlm.nih.gov/geo/geo2r/). ​​We applied the Benjamini–Hochberg procedure to control the false discovery rate (FDR) at 0.05. The results were imported into Excel and the genes of interest were considered differentially expressed when adjusted *p*-value (padj) < 0.05 and logFC>1 (upregulated) or logFC < −1 (downregulated). Interestingly, the expression of some EEC hormone receptors is regulated based on kidney disease, supporting the idea of EEC hormones as another player in gut-kidney communication.

**TABLE 1:** Summary of EECs and their hormonal actions. EEC: enteroendocrine cell; GH: growth hormone; GI: gastrointestinal; AKI: acute kidney injury; ROS: reactive oxygen species; UUO: unilateral ureteral obstruction; IRI: ischemia-reperfusion injury; TSH: thyroid stimulation factor; ADPKD: autosomal dominant polycystic kidney disease; DN: diabetic nephropathy; IEC: intestinal epithelial cell.

EEC	Location in the body/EEC type	Hormone secreted	Hormone receptor	Main effects	Effects on kidney diseases	References
A cells (X-like cells)	Stomach/closed cells	Ghrelin	GHSR	Stimulates the release of GH by the pituitary gland and regulates energetic balance and gastric acid release. Increases appetite, inhibits insulin release, increases GI motility, and renovates gastric and intestinal mucosa	Reduced damage in AKI; Reduced ROS in lesions caused by angiotensin; Attenuated renal fibrosis in UUO	Müller et al. (2015)
						Stengel and Taché (2012)
						Lin and Hsiao (2017)
						Takeda et al. (2006)
						Fujimura et al. (2014)
						Rajan et al. (2012)
						Sun et al. (2015)
		Nesfatin-1 (nucleobindin-2)	Unknown	Regulates food intake, energy consumption, reproduction, GI motility glucose homeostasis, arterial pressure and stress	Anti-inflammatory and antioxidant effects in UUO; anti-apoptotic effects in IRI	Pham et al. (2021)
						Stengel and Taché (2012)
						Prinz et al. (2016)
						Tanida et al. (2015)
						Tezcan et al. (2022)
						Jiang et al. (2015)
D cells	Stomach and Small intestine/Open cells	Somatostatin (SST)	SSTR1/SSTR2A/SSTR2B/SSTR3/SSTR4/SSTR5	Inhibits GH, TSH and GI hormones releasing. Also, inhibits the secretion of insulin, glucagon, gastrin, secretin, cholecystokinin, hydrochloric acid, pepsinogen, and mucosa GI intrinsic factor	Attenuated ADPKD	NCT01377246; Perico et al., 2019
						NCT01354405; Gevers et al., 2015
						NCT02127437; Meijer et al., 2018
						Lecznar et al. (2016)
						Ampofo et al. (2020)
						Garrido-Utrilla et al. (2022)
						Sternini et al. (2008)
						Rezzani et al. (2022)
						Ruggenenti et al. (2005)
						Caroli et al. (2013)
						Suwabe et al. (2021)
G cells	Stomach and Duodenum/Open cells	Gastrin	Cholecystokinin receptor (CCKB or CCK2R)	Stimulate acid secretion	Antiapoptotic effects in IRI; reduced fibrosis in UUO	Hernández et al. (2018)
						Sternini et al. (2008)
						Waldum et al. (2015)
						Liu Y. et al. (2020)
						Gu et al. (2021)
I cells	Small intestine (duodenum and jejunum)/Open cells	Cholecystokinin (CCK)	CCKAR (or CCK1R)/CCKBR (or CCK2R)	Promotes bile releasing, secretion of pancreatic enzymes, and regulation of satiety	Anti-inflammatory effects on kidney macrophages in DN	Hernández et al. (2018)
						Liu et al. (2016)
						Rehfeld (2017)
						Zhang et al. (2021)
						Miyamoto et al., 2012
K cells	Small intestine (Duodenum and Jejunum)/Open cells	Glucose-dependent insulin-releasing polypeptide (GIP)	GIPR	Stimulate insulin secretion, in a state of hypoglycemia, stimulates glucagon secretion, increases the absorption of fatty acids in adipocytes, increases the deposition of triglycerides in subcutaneous adipose tissue, and decreases reabsorption bone	Plausible renoprotection in DN	NCT02237521; Jorgensen et al., 2020
						Khan et al. (2020)
						Hernández et al. (2018)
						Boer et al. (2022)
						Bulum (2022)
						Gasbjerg et al. (2018)
		Xenin	–	Inhibition of food intake, stimulation of insulin release from pancreatic beta cells, and potentiation of the insulinotropic action of GIP	Not investigated	Craig et al. (2018)
						Gault et al. (2015)
L cells	small intestine and large intestine/Open cells	Glucagon-like peptide-1 (GLP-1)	GLP-1R	Stimulates insulin rise, inhibits glucagon secretion and inhibits gastric emptying and food intake	Attenuated renal fibrosis in UUO; reduced ROS in DN; reduced apoptosis in cisplatin AKI; reduced lesions in IRI	Feher (2017)
						Drucker (2018)
						Li et al. (2018)
						Ojima et al. (2013)
						Katagiri et al. (2013)
		Glucagon-like peptide-2 (GLP-2)	GLP-2R	Regulates IEC growth, nutrient absorption and attenuates intestinal inflammation	Not investigated	Sun et al. (2020)
						Kounatidis et al. (2022)
		Glicentin	Unknown	Increase in mucosal growth; inhibition of gastric emptying	Not investigated	Manell et al. (2016)
		Oxyntomodulin	GLP1R/GCGR	It exerts effects on gastric emptying, gastric acid, and intestinal and pancreatic secretion	Not investigated	Cai et al. (2020)
						Druce and Ghatei (2006)
		Peptide YY (PYY)	Y1R/Y2R/Y3R/Y4R/Y5R/Y6R	Reduces postprandial insulin production, promotes satiety, delays gastric emptying, and alters colonic motility	Increased sodium excretion	Meek et al. (2016)
						Playford et al. (1995)
		Insulin-like peptide 5	Relaxin family peptide receptor 4	Regulates insulin secretion, food intake, and colonic motility	Not investigated	Zhang et al. (2020)
M cells	Small intestine (Duodenum and Jejunum)/Open cells	Motilin	MLNR	Increase GI motility, and increase appetite	Not investigated	Hernández et al. (2018)
						Helander and Fändriks (2012)
						Furness et al. (2013)
						Kitazawa and Kaiya (2019)
N cell	Jejunum, ileum, and colon/Open cells	Neurotensin (NTS)	NTSR1/NTSR2/NTSR3	Acts in the control of appetite, and in the regulation of GI motility and secretion	Increased levels associated with decreased renal function	Hernández et al. (2018)
						Engelstoft et al. (2013)
						Guo et al. (2022)
						Mace et al. (2015)
						Osadchii (2015)
						Shulkes et al. (1984)
P cells	Stomach/unknown	Leptin	LEPR (or OBR, or CD295)	Regulates food intake, nutrient absorption, promotes energy homeostasis, and reduces intestinal sugar transport	Associated with renal fibrosis	Cammisotto and Bendayan (2012)
						Mace et al. (2015)
						Jyotaki et al. (2016)
						Mak et al. (2018)
S cells	Small intestine (Duodenum and Jejunum)/unknown	Secretin (SCT)	SCTR	Promote the release of a bicarbonate-rich fluid in the pancreas	Recovered AQP2 expression in DN	Helander and Fändriks (2012)
						Guo et al. (2022)
						Manfredi and Pozzi Mucelli (2016)
						Procino et al. (2014)
Enterochromaffin –cell	Stomach, small and large intestine/Open cells	Serotonin (5-HT)	5-HT1 to 5-HT7 receptor	Regulates hemostasia, intestinal motility, vascular tonus, heart rate, cellular growth and immunoregulatory functions	Associated with neurotoxicity; increased oxidative stress and albuminuria in DN	NCT01165567; Ki et al., 2018
						Engelstoft et al. (2013)
						Xu et al. (2021)
						Helander and Fändriks (2012)
						Herr et al. (2017)
						Takahashi et al. (2002)
						Kobayashi et al. (2008)
		Tachykinin 1 (Tac1, substance P)	TACR1 (or NK1R, or SPR)	Modulation of pain, regulation of emotional behavior, muscle contraction, visceral sensibility, neurogenic inflammation, and peripheral hematopoiesis	Associated with renal inflammation in experimental nephritis; induced M2 macrophage infiltration and reduced tubular necrosis, apoptosis and fibrosis in IRI	Patacchini et al. (2004)
						Nässel et al., 2019
						Beumer et al. (2018)
						Rodionova et al. (2020)
						Kim et al. (2020)
Enterochromaffin - like –cell	Stomach/Open closed	Histamine	H1 receptor	Increase gastric acid	Not investigated	Fakhry et al. (2019)
			H2 receptor			Engelstoft et al. (2013)
			H3 receptor			Lundell et al. (2015)
			H4 receptor			

### 3.1 Ghrelin

Ghrelin is a 28-amino acid (aa) peptide secreted by A cells (also known as X-like cells) predominantly localized in the stomach. Its discovery in 1966 revealed its effect on growth hormone (GH) release stimulation by the pituitary gland through binding to the secretagogue receptor of GH (Müller et al., 2015). However, this peptide also exerts an important role in the regulation of energy balance, gastric acid release, appetite, insulin secretion, gastric motility, and gastric and intestinal mucosal renovation (Lin and Hsiao, 2017). In the kidneys, ghrelin was shown to improve renal function in mice with ischemic acute renal insufficiency through a pathway mediated by Insulin Growth Factor-1, resulting in improved endothelial function (Takeda et al., 2006). A study conducted by Rajan et al. (2012) suggested that its beneficial effect in this model is associated with the activation of the vagus nerve and subsequent negative regulation of inflammatory responses. In fact, ghrelin exhibits the capability to impede the release of a spectrum of cytokines involved in the inflammatory process, mainly IL-1β, IL-6 and tumor necrosis factor-α (TNF-α) through the inhibition of NF-κB expression and activation of the vagus nerve capacity (Wu et al., 2007; Baatar et al., 2011). This latter pathway stimulates the discharge of acetylcholine, which, in turn, curtails the pro-inflammatory impact of macrophages through the activation of α7 nicotinic acetylcholine receptors (α7nAChR) present in these cells (Tanaka et al., 2021). It is noteworthy that macrophages play a central role in driving the inflammatory process associated with kidney disease (Cao et al., 2015). Furthermore, ghrelin also protects against renal damage induced by angiotensin-II by decreasing mitochondrial ROS levels (Fujimura et al., 2014), attenuates renal fibrosis and inflammation on nephropathy induced by unilateral ureteral obstruction (UUO) (Sun et al., 2015). Interestingly, *in silico* data suggest Ghsr mRNA level is downregulated in mice models of UUO (logFC = −1.56 [GSE38117]), suggesting that its renoprotective effect may be not associated with the activation of this receptor.

### 3.2 Nesfatin-1

Nesfatin-1 is an 82-aa polypeptide initially described in the hypothalamus, involved in the regulation of food intake, energy expenditure, reproduction (Pham et al., 2021), GI motility, glucose homeostasis, blood pressure, and stress (Tanida et al., 2015; Prinz et al., 2016). The study by Stengel et al. (2009) indicated that it is also produced in the stomach by A (X-like) cells and is expressed in the brain, duodenum, adipose tissues, heart, and kidney (Chung et al., 2013; Prinz et al., 2016; Sun and Yang, 2018; Pham et al., 2021). Tezcan et al. (2022) described its anti-inflammatory and antioxidant effects, which promote the attenuation of renal fibrosis in rats with UUO. Its ability to improve AKI model ischemia-reperfusion injury (IRI) is also described by downregulating caspase-3, an important protein involved in the process of cell apoptosis while raising Bcl-2 expression levels and reducing the Bax levels, both molecules related to mitochondrial stress and intrinsic-pathway of cellular apoptosis (Jiang et al., 2015). Additional research has indicated that nesfatin-1 exerts an anti-inflammatory function by suppressing inducible nitric oxide synthase and cyclooxygenase, resulting in the subsequent decrease of nitric oxide and prostaglandin E2 levels (Jiang et al., 2020). Moreover, this hormone acts to inhibit NF-κB (Xu and Chen, 2020), effectively impeding the transcription of pro-inflammatory genes, consequently reducing renal inflammation (Zhang and Sun, 2015). Although its receptor has not yet been characterized, research indicates that it may be a GPCR (Brailoiu et al., 2007; Ishida et al., 2012), such as GPR12 (Pham et al., 2021).

### 3.3 Somatostatin (SST)

SST is a peptide with two active isoforms, a short one (14-aa) that acts mainly in the brain, and a long-form (28-aa) that acts in the GI tract (O'Toole and Sharma, 2023). This hormone is secreted throughout the central nervous system, in the retina, in peripheral neurons, and in the pancreatic islets of Langerhans. However, 65% of the body’s total SST is produced in the GI tract (Rai et al., 2015; Messchendorp et al., 2020), specifically by D cells harboring the stomach and the duodenum, with smaller quantities found throughout the GI tract (Garrido-Utrilla et al., 2022; Rezzani et al., 2022). It is less common in the colon and rectum (Gunawardene et al., 2011). Thus, it has a wide range of activity, such as inhibiting the release of GH, thyroid-stimulating hormones, and GI hormones. Furthermore, it can inhibit the secretion of insulin, glucagon, gastrin, secretin, cholecystokinin, hydrochloric acid, pepsinogen, and intrinsic factor of the GI mucosa (Lecznar et al., 2016; Ampofo et al., 2020).

The kidneys can also produce this hormone, but in smaller amounts, and they express SST receptors (Messchendorp et al., 2020). Nonetheless, SST has a short half-life (1–3 min), which limits its therapeutic potential. Therefore, new analogs are being developed to overcome this limitation. These analogs have shown positive effects in renal therapy, particularly in autosomal dominant polycystic kidney disease (ADPKD), a slowly progressive disease that culminates in ESRD (Ruggenenti et al., 2005; Caroli et al., 2013; Suwabe et al., 2021). Treatment with the long-acting SST analog octreotide in ADPKD has slowed kidney growth and delayed progression to CKD (Perico et al., 2019). Furthermore, lanreotide, another SST analog, has reduced polycystic kidney volumes and decreased symptoms in patients with ADPKD (Gevers et al., 2015). However, the use of lanreotide in the treatment of ADPKD did not delay the decline in renal function over 2.5 years of follow-up, which does not support its use for the treatment of advanced-stage ADPKD (Meijer et al., 2018). To the best of our knowledge, octreotide, concerning AKI, had its possible renoprotective effect evaluated only in Hepatic ischemia and reperfusion injury (HIR), where it was shown to reduce kidney damage after HIR due to deactivation of the protein kinase B (Akt)-mammalian target of rapamycin (mTOR) pathway and reduction of renal inflammation and apoptosis (Sun et al., 2017). These data open up a field of research that deserves to be better investigated. Our *in silico* analysis shows that only Sstr3, the SST receptor, is downregulated in a murine model of IRI (logFC = −1,04 [GSE39548]).

### 3.4 Gastrin

Gastrin is a peptide consisting of 17-aa produced in the G cells of the stomach and duodenum (Hernández et al., 2018). Its action occurs after binding to the cholecystokinin GPCR called CCK-B receptor (CCK-BR) in Enterochromaffin-like cells, thus promoting gastric acid secretion (Waldum et al., 2015). Despite being an intestinal hormone, its effect on distant organs such as the kidney is suggested based on the existence of CCK-BR in this organ (Liu et al., 2016). In fact, in an IRI model, gastrin attenuated renal injury by exerting an anti-apoptotic effect by inhibiting the phosphoinositide-3 kinase (PI3K)-Akt pathway, a crucial pathway involved in the recruitment of inflammatory factors (Liu C. et al., 2020). Recent data also demonstrate that the use of subcutaneous gastrin infusion improves interstitial fibrosis and UUO through activation of peroxisome proliferator-activated receptor alpha, reduction of apoptosis of renal tubular cells, and reduction of renal inflammation (Gu et al., 2021).

### 3.5 Cholecystokinin (CCK)

In 1928 Ivy and Oldberg (1928) described CCK, a peptide that exists in multiple molecular forms after cell-specific post-translational processing, with the 33-aa form (CCK-33) in plasma being predominant in humans, and CCK-8 in the brain (Zeng et al., 2020). This hormone is secreted by I cells in the proximal intestine (duodenum and jejunum) (Hernández et al., 2018) and shares the same receptors with gastrin, the CCKA receptor (CCKAR), and the CCKB receptor (CCKBR). CCK in the sulfated form has a higher affinity for CCKAR than gastrin, while CCKBR binds and responds to gastrin or CCK with similar affinity, without distinction between sulfated and non-sulfated forms (Liu et al., 2016). Its function is diverse, such as promoting gallbladder emptying, secretion of pancreatic enzymes (Rehfeld, 2017), and regulation of satiety (Zhang et al., 2021). Although the effect of this hormone on the kidneys remains little explored, the study conducted by Miyamoto et al. (2012) demonstrated that CCK exerts a renoprotective effect in diabetic animals through anti-inflammatory actions on the macrophage. This hormone is also capable of stimulating the vagus nerve and releasing acetylcholine, which binds to α7nAChR on macrophages, thereby instigating an anti-inflammatory effect by inhibiting the activation of cytokines such as TNF-α and IL-6 (Luyer et al., 2005). Furthermore, *in silico* data demonstrate that the Cckar in samples from the kidneys of mice is downregulated in DN (logFC = −1,50 [GSE197699]), UUO (logFC = −3,11 [GSE87212] and logFC = −1,40 [GSE42303]), cisplatin-induced nephropathy (logFC = −2,74 [GSE106993]), sepsis (logFC = −1,88 [GSE60088]), and lupus nephritis (logFC = −1,37 [GSE86425] and logFC = −1,16 [GSE32583]). Also, Cckbr in the murine model of UUO is downregulated (logFC = −1,45 [GSE38117]). Since overall Cckar expression is downregulated in different kidney diseases, it is plausible to speculate that Cckar might be an important receptor to help maintain kidney homeostasis.

### 3.6 Glucose-dependent insulin-releasing polypeptide (GIP)

In 1970, Brown et al. (1970) isolated a 42-aa peptide for the first time, which was secreted by K cells primarily located in the stomach and proximal intestine (duodenum and jejunum). This peptide was found to inhibit gastric acid secretion and was initially named gastric inhibitory polypeptide. However, due to its ability to postprandially stimulate insulin secretion in a glucose-dependent manner, it was later renamed glucose-dependent insulinotropic polypeptide (Khan et al., 2020). Apart from its insulin-stimulating effects, GIP, during hypoglycemia, stimulates glucagon secretion (Gasbjerg et al., 2018), enhances the absorption of fatty acids in adipocytes, increases the deposition of triglycerides in subcutaneous adipose tissue (Boer et al., 2022) and decreases bone reabsorption (Gasbjerg et al., 2018). Similar to GLP-1, GIP is rapidly degraded by dipeptidyl peptidase 4 (DPP-4), resulting in a short half-life of a few minutes (Idorn et al., 2014). Both hormones are classified as incretins because of their ability to increase insulin secretion, and there is evidence suggesting a potential renoprotective action of GIP in DN (Bulum, 2022). Indeed, individuals with ESRD exhibit reduced responsiveness to incretin hormones, leading to elevated glucagon levels and a higher prevalence of glucose intolerance in these patients (Jorgensen et al., 2020). Interestingly, GIP has been shown to exert anti-inflammatory effects by reducing plasma levels of IL-1β, IL-6 and TNF-α in mice, which are associated with increased severity of kidney disease. Furthermore, GIP has shown promise in improving determinants of nephropathy, such as diabetes and hypertension (Bulum, 2022). Notably, GIP exhibits the capacity to reduce macrophage infiltration, a potentially positive attribute in the context of renal inflammatory diseases (Nagashima et al., 2011). The pathways activated by GIP warrant thorough investigation, as do the effects of GIP receptor agonists in kidney disorders. This is particularly pertinent due to the observed upregulation of GIP receptor mRNA in the kidneys of mice submitted to UUO (LogFC = 5.68 [GSE87212]).

### 3.7 Xenin

Xenin is a 25-aa GI hormone produced and secreted by K cells, but it does not have, so far, a known specific receptor (Craig et al., 2018). Although studies suggest its ability to interact with the neurotensin receptor, others have already demonstrated that its effects are independent of neurotensin receptor activation (Craig et al., 2021). Xenin stimulates insulin release from pancreatic beta cells, inhibits food intake, and potentiates the insulinotropic action of GIP (Gault et al., 2015). This hormone has also been identified to be produced in other organs such as the hypothalamus, heart, liver, pancreas, testes, skin, and kidney (Craig et al., 2021), but its effect on the kidneys has not yet been reported. Indirect indications suggest the potential benefits on kidney health that can be derived from the use of xenin. This hormone is able to reduce the expression of NF-κB and Toll-like receptor 4 (TLR4) (Denver et al., 2018). Dysregulated NF-κB activation is one of the main causes of inflammatory kidney diseases (Zhang and Sun, 2015), xenin’s ability to modulate this pathway is particularly promising. Furthermore, the absence of TLR4 prevents the activation of the p38 mitogen-activated protein kinase (MAPK) pathways, which in turn reduces the production of inflammatory cytokines, such as TNF-α, mitigating kidney damage (Zhang et al., 2008).

### 3.8 Glucagon-like peptide-1 (GLP-1)

GLP-1 is an endogenous incretin derived from post-translational processing of the proglucagon gene, whose sequence and polypeptide chain gives rise beyond the GLP-1 to GLP-2, Glicentin, Oxyntomodulin, PYY, and Insulin-like peptide 5 (Gunawardene et al., 2011; Nauck and Meier, 2018; Simsir et al., 2018). GLP-1 has several isoforms, with GLP-1 (7–36) being the most common and biologically active (May et al., 2019). This hormone is secreted by L cells expressed in the small and large intestines in response to food ingestion (Manandhar and Ahn, 2015), promoting glycemic control by stimulating an increase in insulin and inhibiting glucagon secretion. Additionally, it can inhibit gastric emptying and food intake (Drucker, 2018). As this is a short-lived hormone, most of the evidence supporting the relationship between EECs-releasing hormones and the kidneys comes from modified molecules based on the structural basis of GLP-1. Liraglutide, for example, a GLP-1 analog with a longer half-life, can attenuate UUO-induced renal fibrosis through activation of GLP-1 receptor (GLP-1R) and activation of TGF-β1/Smad3 and extracellular signal-regulated kinases (ERK) 1/2 signaling pathways, thereby decreasing extracellular matrix secretion and deposition (Li et al., 2018). Our *in silico* analyses of public data show a reduction in GLP-1R expression in the kidney samples from mice submitted to UUO (logFC = −3,27 [GSE87212] and logFC = −1,02 [GSE38117]), supporting the use of a GLP-1R agonist as an interesting therapeutic alternative in remodeling of renal fibrosis. Furthermore, in diabetic rat models, exendin-4, a GLP-1-like peptide capable of activating GLP1-R, was able to protect against the onset and progression of DN by inhibiting the formation of ROS (Ojima et al., 2013), and its effect on reducing cisplatin-induced kidney injury and apoptosis has also been demonstrated (Katagiri et al., 2013). Part of these effects is associated with the ability of GLP-1 to positively regulate the production of cyclic adenosine monophosphate (cAMP), which stimulates protein kinase A (PKA) activity. This intricate mechanism leads to the suppression of oxidative renal damage, a principal contributor to DN (Bulum, 2022). Another widely used alternative is the use of inhibitors of the DPP-4 enzyme, which cleaves and inactivates GLP-1, such as sitagliptin, which has been shown to protect rat kidneys from acute IRI through upregulation of GLP-1 and GLP-1R (Chang et al., 2015). Nonetheless, when comparing strategies, the use of a GLP-1R agonist has shown better results compared to a DPP-4 inhibitor among patients with type 2 diabetes and advanced CKD (Chen et al., 2022). Simultaneously, research findings propose an indirect correlation between bariatric and the improvement of kidney diseases, especially DN, in a mechanism that involves GLP-1. Following bariatric surgery, GLP-1 serum levels are elevated due to the heightened secretion of bile acids on digestive juice, a process that subsequently stimulates the secretion of GLP-1 by EECs. Elevated incretin levels serve to lower glycemia and improve satiety, resulting in a better prognosis of diabetes (Han et al., 2015; Wang et al., 2019), and by extension, DN as well. These insights introduce an additional layer of potential benefit associated with GLP-1 within the context of the gut-kidney axis, reinforcing the pivotal role of EECs in this interplay.

### 3.9 Glucagon-like peptide-2 (GLP-2)

GLP-2 is a 33-aa peptide hormone that acts through interaction with the GLP-2 receptor (GLP-2R). It is distinct from GLP-1 and plays a role in regulating cell growth in the intestinal epithelium, attenuating intestinal inflammation while aiding nutrient absorption (Sun et al., 2020; Kounatidis et al., 2022). Due to its inhibition by DPP-4, the reported effects of GLP-1 on renal function when using DPP-4 inhibitors may be also attributed to GLP-2, suggesting a possible synergy between them. Teduglutide, a GLP-2 analog resistant to degradation, has been shown to reduce the amplitude of daily fluctuations in water balance, which is associated with the beneficial effects of GLP-2 on kidney function (Jeppesen et al., 2009). However, studies on the use of this molecule to treat or prevent kidney diseases are still scarce. The study conducted by Villanueva et al. (2010) indicates that GLP-2 has the capacity to enhance the expression of multidrug resistance-associated protein 2 (Mrp2) in the jejunum. This finding consequently raises the prospect of investigating the GLP-2-mediated modulation of Mrp2 expression within the kidneys. Mrp2 is an important ATP-binding cassette transporter involved in the detoxification process. The augmentation of its gene expression yields significant benefits, particularly in drug-mediated kidney diseases such as acetaminophen-induced nephrotoxicity, through of the upregulation of the Nrf2-Mrp2/4 pathway (Zhang et al., 2022).

### 3.10 Glicentin

Glycentin is a 69-aa hormone that participates in the regulation of GI motility, gastric acid secretion, and insulin secretion (Manell et al., 2016). Despite the important role of the kidney in degrading glicentin has long been described (Lopez-Novoa et al., 1986), there is no receptor identified for glicentin and its role in kidney disease remains unexplored. However, the involvement of GLP-1R in this process cannot be ruled out, since the use of a selective GLP-1R antagonist (exendin-(9–39)) promoted a reduction in glicentin activity (Ayachi et al., 2005; Raffort et al., 2017). Nevertheless, the precise nature of GLP1R’s participation remains uncertain. Approximately 1/3 of glicentin may have been metabolized into oxyntomodulin, a hormone known to interact with GLP-1R (Cai et al., 2020). Therefore, a comprehensive understanding of the pathways underpinning the response to glicentin remains elusive.

### 3.11 Oxyntomodulin

Oxyntomodulin, a peptide hormone composed of 37-aa, was isolated from porcine jejunum-ileum in 1982 by Bataille et al. (1982). It is a product derived from the cleavage of glicentin, which acts through the GLP-1R and glucagon receptor (GCGR) (Cai et al., 2020), exerting effects on gastric emptying, gastric acid, and intestinal and pancreatic secretion (Druce and Ghatei, 2006). The therapeutic potential of oxyntomodulin in kidney diseases is still poorly explored. However, recent research has indicated promising outcomes. The utilization of a prolonged-acting derivative of oxyntomodulin has demonstrated its ability to improve kidney injury through the activation of both GLP-1 and glucagon receptors (Huang et al., 2021). This suggests that the underlying pathways of oxyntomodulin’s effect might mirror those discussed in the GLP-1 section. Moreover, its dual agonism GLP1R/GCGR receptors may potentiate these effects (Pocai, 2014). Furthermore, it has been demonstrated that oxyntomodulin is capable of inhibiting the activation of the NF-κB pathway in the spinal cord (Zhang et al., 2019). It is noteworthy that during renal inflammatory processes, immune cells such as neutrophil, monocytes, and macrophages are recruited. The stimulation of pro-inflammatory gene production is facilitated by the activation of transcription factors, with NF-κB being the most predominant (White et al., 2020). Of particular interest, there is a report indicating that the selective activation of GCGR by oxyntomodulin boosts the concentration of fibroblast growth factor-21 (FGF-21) mRNA in the liver, along with plasma FGF-21 levels (Pocai, 2014). FGF-21 is a molecule that interconnects the liver-brain-kidney axis and promotes renal gluconeogenesis. Intriguingly, this process may contribute to the acceleration of the renal cell carcinoma progression (Li et al., 2023).

### 3.12 Peptide YY (PYY)

PYY 1-36 is subjected to the action of the DPP-4 enzyme, converting it into PYY3-36. This active form binds to specific receptors (Y1R/Y2R/Y3R/Y4R/Y5R/Y6R), reducing postprandial insulin production, promoting satiety, delaying gastric emptying, and affecting colonic motility (Meek et al., 2016). In rats, PYY infusion increased mean arterial pressure and decreased renal plasma flow without significantly altering glomerular filtration rate and sodium excretion (Blaze et al., 1997). In normal patients, PYY infusion resulted in a reduction in glomerular filtration rate, plasma renin activity, and aldosterone levels, with an increase in sodium excretion. This suggests that the PYY receptor could be a potential therapeutic target for the treatment of patients with sodium overload (Playford et al., 1995). In contrast, no renal impairment has been reported in animal models deficient in neuropeptide Y (NPY) or its receptor (Winaver and Abassi, 2006). NPY is a PYY-like peptide provided by enteric neurons, which shares an affinity with PYY for Y1, Y2, and Y5 receptor subtypes. PYY3-36 has a greater affinity with Y2 (Holzer et al., 2012). Nonetheless, further evaluation is required to determine its effect on kidney diseases. The NPY1R gene is upregulated in kidney samples of lupus nephritis in humans (logFC = 1,36 [GSE32591]). In mice, the Npy6r gene is upregulated in UUO (logFC = 3,1629 [GSE87212] and logFC = 2,04 [GSE38117]), while the Npy4r (logFC = −1,07 [GSE38117]) gene is downregulated in UUO. Presumably, PYY may act in different ways in the kidneys, depending on the activated receptor. Thus, future studies to evaluate the impact of different PYY receptors on the kidney are necessary.

### 3.13 Insulin-like peptide 5

Insulin-like-5 (INSL5) is a 135-aa peptide hormone that regulates insulin secretion, food intake, and colonic motility. Its receptor, Relaxin/Insulin Like Family Peptide Receptor-4 (RXFP4), has been extensively studied as a possible pharmacological target for the treatment of constipation, anorexia, and obesity (Zhang et al., 2020), but its use in kidney diseases remains unexplored. The administration of the INSL5 in bone-marrow-derived macrophages from C57BL/6 mice led to the downregulation of IL-6, IL-1β and TNF-α expression (Hechter et al., 2022). Thus, this finding suggests that INSL5 exerts an anti-inflammatory effect on the kidneys.

### 3.14 Motilin

In 1971, Brown et al. (1971) isolated a polypeptide with 22-aa residues from the mucosa of the upper portion of the small intestine of pigs, which was called Motilin. This hormone is secreted by M cells present in the small intestine, especially in the duodenum and jejunum. Motilin’s primary function is to induce intestinal motility through the stimulation of the motilin receptor (MLNR) (Furness et al., 2013; Kitazawa and Kaiya, 2019). A motilin serum concentration is increased in mice and patients with CKD (Wu et al., 2017; Ali et al., 2020), however, whether this hormone has a negative or positive effect on kidney disorders has yet to be evaluated.

### 3.15 Neurotensin (NTS)

NTS is a 13-aa peptide that was initially isolated from the bovine hypothalamus and described as a hypotensive peptide by Carrawaye and Leeman (1973). Currently, it is known that this hormone is secreted by N cells located in the jejunum, ileum, and colon (Mace et al., 2015; Guo et al., 2022). After the discovery of its expression in the brain and GI tract, there was an extensive investigation of its role in endocrine functions, pain modulation, and pathogenesis of mental disorders, appetite control, as well as its regulation of GI motility and secretion (Osadchii, 2015). However, little is known about its role in kidney disease. Limited data suggest that circulating levels of NTS increase with the deterioration of renal function (Shulkes et al., 1984), which is likely due to the renal metabolism of this hormone, since the mRNA expression of NTS in mice with diabetic kidney disease does not significantly increase (Tönjes et al., 2020). Valuable insights from an intestinal inflammation model revealed that NTS can trigger the release of the pro-inflammatory cytokine IL-8 in an ERK or NF-κB pathway-dependent manner. Furthermore, in NTS knockout mice showcased reduced macrophage infiltration, along with diminished activation of NF-κB and IL-6 in the absence of NTS (Mustain et al., 2011; Ye et al., 2016). We analyzed the gene expression in a variety of kidney diseases, and only in a murine model of renal fibrosis UUO, the expression of Ntsr1, which encodes the receptor for NTS, upregulated (LogFC = 4,2604 [GSE87212]). These findings collectively suggest a potential scenario where increased Ntsr1 activation occurs during renal fibrosis. This might be linked to higher plasma concentration of NTS and increased renal expression of Ntsr1, leading to a pro-inflammatory state. Further, *in vivo*, experiments are required to evaluate the effect of this receptor on renal fibrosis.

### 3.16 Leptin

Leptin, a 167-aa peptide, was initially described in adipose tissue. It is now recognized that the gastric mucosa is an important production site for this hormone, which is secreted by nutrient-stimulated P cells (Cammisotto and Bendayan, 2012; Mace et al., 2015). Activation of the leptin receptor (LEPR) regulates food intake, nutrient absorption, promotes energy homeostasis, and can inhibit sodium-glucose transporter-1 (SGLT1) in the intestine, thereby reducing intestinal sugar transport (Jyotaki et al., 2016). In murine models of uremic cachexia and renal fibrosis, the use of leptin antagonists significantly reduced levels of TGF-β1 mRNA, which can be coactivated by leptin to promote the development of muscle and renal fibrosis (Mak et al., 2018). Indeed, Lepr is upregulated in the context of UUO (logFC = 2,18 [GSE38117] and logFC = 1,42 [GSE87212]). Activation of Lepr triggers the activation of the MAPK family of signaling pathways, particularly the non-canonical TGF-β1/MAPK signaling pathway in the progression of renal fibrosis (Pérez-Pérez et al., 2020; Cheng et al., 2021). However, in humans, LEPR is downregulated in DN (logFC = −1,37 [GSE30122]). The exact role of CKD remains elusive.

### 3.17 Secretin

Secretin is a 27-aa peptide that is secreted by S cells in the duodenum and jejunum. It acts through the secretin receptor (SCTR) to stimulate the release of a bicarbonate-rich fluid in the pancreas (Manfredi and Pozzi Mucelli, 2016; Guo et al., 2022). Studies using knockout animals for SCTR have shown that these animals may exhibit mild polydipsia and polyuria (Chu et al., 2007). Intriguingly, in polycystic kidney disease, the absence of this receptor did not affect the severity of the disease (Wang et al., 2012). The pharmacological use of this hormone in kidney diseases remains little explored. In models of nephrogenic diabetes insipidus linked to the X chromosome, secretin has been found to be effective in rescuing the negative regulation of the Aquaporin 2 protein. When combined with fluvastatin, it increases conservation/solute reabsorption, providing a suitable osmotic gradient for water reabsorption (Procino et al., 2014). *In silico* data indicate that Sctr is upregulated in mice with UUO (LogFC = 3,20 [GSE181380] and logFC = 1,93 [GSE38117]) and in humans with IgAN (logFC = 1,02 [GSE116626]). Research conducted in different contexts can provide valuable insights into the potential mechanisms and pathways through which secretin operates in the kidney. An illustrative example can be found in liver fibrosis, where secretin has been shown to initiate the upregulation of TGF-β1 signaling (Kyritsi et al., 2023). This signaling cascade is well-known for its ability to induce renal fibrosis in murine models of UUO, concurrently triggering anti-inflammatory reactions and pro-fibrotic responses. This intricate dance is facilitated by the interaction between *β*-catenin and forkhead box, as well as T Cell factor (Tang et al., 2021). Further evaluation of its effects *in vivo* is needed, with special attention on renal fibrosis and immune-mediated renal disease such as IgAN.

### 3.18 Serotonin

Serotonin, also known as 5-hydroxytryptamine (5-HT), is a hormone whose 90% of its production occurs in Enterochromaffin cells (Xu et al., 2021) in the stomach, small and large intestine, with the duodenum being the predominant site (Helander and Fändriks, 2012). Its functions include the regulation of hemostasis, intestinal motility, vascular tone, heart rate, cell growth, and immunoregulatory processes (Herr et al., 2017). To date, a limited number of studies demonstrate that increasing the plasma concentration of serotonin can have a negative effect on kidney disease (Takahashi et al., 2002; Kobayashi et al., 2008; Agarwal, et al., 2017). The use of the pesticide dichlorvos increased serotonin levels in the kidneys of mice, which is associated with an increase in free radicals (Agarwal et al., 2017). However, it remains unclear whether the renotoxic effect is directly caused by serotonin. In models of diabetic kidney disease, the 5-HTR antagonist sarpogrelate was able to reduce albuminuria, suppress oxidative stress and improve glomerular endothelial function (Takahashi et al., 2002; Kobayashi et al., 2008). However, it did not prevent contrast-induced nephropathy in CKD patients (Ki et al., 2018). The Htr2b gene, which encodes the receptor for serotonin, is upregulated in mice with UUO (LogFC = 3,1697 [GSE87212] and logFC = 1,28 [GSE38117]), as well as in humans with IgAN (logFC = 1,57 [GSE116626] and logFC = 1,76 [GSE93798]), lupus nephritis (logFC = 1,90 [GSE32591]) and DN (logFC = 1,84 [GSE30122]). Conversely, Htr2a in mice is upregulated in UUO (LogFC = 3,26 [GSE87212]), while Htr3a (logFC = −2,32), Htr3b (logFC = −1,11) and Htr4 (logFC = −1,07) are downregulated in the murine model of UUO (GSE38117) and Htr2c is downregulated in the rat model of AKI induced by cisplatin (logFC = −1,31 [GSE37133]). A substantial body of evidence supports the pro-inflammatory influence of serotonin. This is demonstrated by cytokine production in monocytes following LPS stimulation, as well as observations of reduced inflammation in arthritis associated with serotonin depletion (Harbuz et al., 1998; Talsma and Woldorff, 2005; Pelletier and Siegel, 2009). However, contrasting reports suggest that activation of 5-HT2A receptors might actually suppress TNF-α responses, subsequently resulting in the reduction of IL-6 levels and hindering NF-κB translocation (Yu et al., 2008). Given these divergent findings, it becomes necessary to delve into the effects of serotonin on various kidney conditions. Furthermore, there is a need to investigate which specific receptors are involved in these intricate processes. Clarifying these dynamics will significantly contribute to our understanding of the interplay between serotonin and kidney health.

### 3.19 Tachykinin

Tachykinin, also called substance P, is an 11-aa peptide produced in the brain and intestine by Enterochromaffin cells (Beumer et al., 2018; Nässel et al., 2019). It has a broad range of effects, including the modulation of pain perception, regulation of emotional behavior, muscle contraction, visceral sensitivity, induction of neurogenic inflammation, and regulation of hematopoiesis at the peripheral level (Patacchini et al., 2004). In the kidneys, tachykinin’s effect has been described in the control of water and electrolyte excretion through activation of the tachykinin receptor 1 (TACR1) also known as substance P receptor (SPR) or neurokinin 1 receptor (NK1R), as well as the renal sympathetic nerve (Ding Yuan and Couture, 1997). However, in an experimental nephritis model, tachykinin was associated with renal inflammation due to the recruitment of dendritic cells that express NK1R (CD11c+NK1R+), macrophage infiltration, and increased renal expression of chemokines (Rodionova et al., 2020). The ability of tachykinin to activate the PI3K-Akt signaling pathway in dendritic cells is also reported (Janelsins et al., 2009). Furthermore, inhibiting this pathway has demonstrated efficacy in diminishing the fibrotic process by curtailing the release of pro-inflammatory cytokines and extracellular matrix deposition (Xiang et al., 2022). The exact mechanism through which G proteins activate PI3K remains unclear. PI3K is capable of converting phosphatidylinositol 4,5-bisphosphate (PIP2) to phosphatidylinositol-3,4,5-triphosphate (PIP3), which activates Akt. Akt activation prompts fibroblast proliferation and activation, pivotal elements in the progression of renal fibrosis (Kim et al., 2021; An et al., 2022). Conversely, the phosphatase and tensin homolog (PTEN) counters this process by dephosphorylating PIP3 (Garcia-Recio and Gascón, 2015). Interestingly, PTEN deficiency is associated with heightened macrophages infiltration and an elevated occurrence of renal fibrosis (Na et al., 2022). In the IRI model of renal injury, tachykinin induced macrophage infiltration after 4 weeks. However, it was able to shift the polarization of intrarenal macrophages from CCR7+M1 macrophages to CD206+M2 macrophages in injured kidneys, preserving kidney size, normal tubular structures, alleviating tubular necrosis, inflammation, apoptosis, and tubulointerstitial fibrosis (Kim et al., 2020). Interestingly, our *in silico* analysis indicates that in this model, the Tacr1 gene, which encodes the receptor for the hormone tachykinin, is downregulated (logFC = −1,07 [GSE39548]). This observed beneficial renal effect may be associated with increased activation of this receptor in the kidneys. In addition, Tacr3 is downregulated in UUO (logFC = −1,16 [GSE38117]). It is worth mentioning that tachykinin-related peptide has been shown to regulate stress resistance, insulin production, and signaling in *Drosophila* renal tubules (Söderberg et al., 2011), but this effect has not yet been evaluated in mammals.

### 3.20 Histamine

Histamine is produced and secreted in the stomach by Enterochromaffin-like cells, which regulate gastric acid production by binding to histamine-2 (H2) receptors on parietal cells (Lundell et al., 2015). Although this receptor is present in the kidneys and its activation influences urinary dilution and tubular reabsorption in the canine kidney (Radke et al., 1985), most studies assessing its renal impact have been conducted through infusion. Other cells, such as mast cells, basophils, lymphocytes, and neurons in the central nervous system, can also produce histamine (Mahdy and Webster, 2011). Interestingly, our *in silico* analyses indicate that genes encoding histamine receptors exhibit alterations during renal disorders. Of interest, Hrh4 is downregulated in UUO (logFC = −1.03 [GSE38117]) and Hrh3 is downregulated in IRI (logFC = −1.26 [GSE39548] and LogFC = −2.87 [GSE181380]), models of chronic and acute kidney disease, respectively. Both receptors are coupled to the Gi/o protein, and their activation is responsible for inhibiting forskolin-induced cAMP formation (Akdis and Simons, 2006). This may indicate a possible adverse effect of histamine because cAMP promotes PKA activation. This PKA activation, in turn, blocks gene transcription of proinflammatory cytokines mediated by TGF-β signaling. In essence, cAMP exerts an antifibrotic effect on renal fibrosis, which would be beneficial in a fibrotic model such as UUO (Schinner et al., 2015). Furthermore, in the I/R model, cAMP stimulation is able to reduce the severity of kidney damage, reduce the accumulation of neutrophils and reduce the production of TNF-α (Mizutani et al., 2005). Also, Hrh1 shows upregulation in the UUO mouse model (logFC = 1.84 [GSE38117] and LogFC = 2.59 [GSE87212]). This receptor is coupled to Gq/11 and its activation stimulates the NF-κB pathway (Akdis and Simons, 2006). These observations collectively shed light on the interactions between histamine receptors within the kidney, unveiling potential pathways through which histamine influences renal disorders and fibrosis.

## 4 Clinical relevance of EEC hormones

To date, despite the therapeutic potential of enteroendocrine hormones in the treatment of kidney diseases, only Somatostatin, GLP-1 and Serotonin have consolidated results in clinical trials (Table 2). Three randomized clinical trials (Gevers et al., 2015; Meijer et al., 2018; Perico et al., 2019) assessed the impact of Somatostatin analogues such as Lanreotide and Octreotide on Autosomal Dominant Polycystic Kidney Disease (ADPKD). The administration of lanreotide at a dose of 120 mg intramuscularly every 28 days, when compared to conventional treatment, did not exhibit a delay in the decline in renal function over a 2.5 years of follow-up period (Meijer et al., 2018). However, this same therapeutic regimen did reduce the volume of polycystic kidneys, as well as the symptoms of ADPKD (Gevers et al., 2015). Furthermore, the use of 40 mg of Octreotide every 28 days (administered as two 20 mg intragluteal injections for 3 years) managed to delay renal growth and the progression of patients to end-stage renal disease (Perico et al., 2019). Positively, these medications have a long half-life, obviating the need for daily administration to achieve a therapeutic effect. Besides, they are widely employed in clinical practice across various conditions and boast well-established pharmacokinetic and pharmacodynamic profiles, including patients with kidney impairments. Thus, these medications could serve as adjuncts to renal therapy.

**TABLE 2:** Summary of Clinical studies involving hormones secreted by EECS in kidney disease. ADPKD, autosomal dominant polycystic kidney disease; CKD, chronic kidney disease.

NCT Number/Disease	Interventions	Phase/Follow-up period	Results
NCT0137724/ADPKD; Perico et al. (2019)	Drug: Octreotide (Somatostatin analogue)40 mg every 28 days (in two intragluteal 20 mg injections) for 3 yearsControl: Saline solutionAt the same volume of study drug every 28 days (in two intragluteal injections) for 3 years	Phase 3/3 years	In advanced-stage ADPKD, octreotide slowed kidney growth and delayed progression to end-stage renal disease, particularly in stage 4 CKD.
NCT0135440/Polycystic kidney disease; Gevers et al. (2015)	Drug: Lanreotide (Somatostatin analogue)120 mg every 28 days intramuscularControl: Standard care (blood pressure control, a sodium-restricted diet, and antihypertensive agents)	Phase 3/24 weeks	Lanreotide is able to reduce polycystic kidney volumes as well as symptoms in ADPKD. Furthermore, the estimated glomerular filtration rate is sharply reduced after initiation of lanreotide, stabilized thereafter and decreases again after withdrawal
NCT0212743/ADPKD; Meijer et al. (2018)	Drug: Lanreotide (Somatostatin analogue)120 mg, subcutaneously, once every 4 weeksControl: Standard care (blood pressure control, a sodium-restricted diet, and antihypertensive agents)	Phase 3/120 weeks	Treatment with lanreotide compared to standard care did not delay the decline in renal function over 2.5 years of follow-up. These findings do not support the use of lanreotide for the treatment of advanced autosomal dominant polycystic kidney disease
NCT01620489/Diabetes Mellitus, Type 2; Davies et al. (2016)	Drug: Liraglutide (GLP-1 analogue)1.8 mg administered once daily subcutaneously as add-on to the subject’s stable pre-trial oral antidiabetic drug (OAD) and/or insulin regimenControl: PlaceboAdministered once daily subcutaneously as add-on to the subject’s stable pre-trial oral antidiabetic drug (OAD) and/or insulin regimen	Phase 3/26 weeks	Liraglutide did not affect renal function and demonstrated better glycemic control, with no increase in hypoglycemia risk but with higher withdrawals due to GI adverse events than placebo in patients with type 2 diabetes and moderate renal impairment
NCT01621178/Chronic Kidney Disease; Tuttle et al. (2018)	Drug: Dulaglutide (GLP-1 receptor agonist)0.75 mg administered once weekly subcutaneouslyControl: Insulin glargine administered SC	Phase 3/52 weeks	In patients with type 2 diabetes and moderate-to-severe chronic kidney disease, once-weekly dulaglutide produced glycaemic control similar to that achieved with insulin glargine, with reduced decline in eGFR. Dulaglutide seems to be safe to use to achieve glycaemic control in patients with moderate-to-severe chronic kidney disease
NCT02827708/Diabetes Mellitus, Type 2; Mosenzon et al. (2019)	Drug: Semaglutide (GLP-1 receptor agonist)14 mg - Oral administration once dailyControl: PlaceboOral administration once daily	Phase 3/26 weeks	Semaglutide had greater effects than placebo, resulting in a greater reduction in HbA1c and body weight in patients with type 2 diabetes and moderate renal failure
NCT01165567/Diabetes Mellitus, Type 2; Ki et al. (2018)	**Drug**: sarpogrelate (Serotonin receptor antagonist) sarpogrelate, 300 mg/day,duration: 4 weeks**Control**: Saline solution	Phase 4/4 weeks	The use of sarpogrelate did not demonstrate a renoprotective effect against acute kidney injury caused by contrast

Conversely, Liraglutide, Dulaglutide and Semaglutide (GLP-1 receptor agonists) also demonstrated promising outcomes. Subcutaneous administration of 1.8 mg of Liraglutide once daily as a complement to oral antidiabetic medication improved glycemic control. However, its use has been associated with gastrointestinal adverse events in patients with type 2 diabetes and moderate renal failure (Davies et al., 2016). In separate clinical trials, the use of 0.75 mg of Dulaglutide administered once weekly subcutaneously (Tuttle et al., 2018) and 14 mg of Semaglutide once daily orally (Mosenzon et al., 2019) led to significant reductions in HbA1c and body weight in patients with type 2 diabetes and moderate renal failure. These findings affirm the safety and efficacy of GLP-1 agonists in patients with moderate to severe CKD, with few episodes of hypoglycemia, and suggest they can facilitate improved glycemic control. This latter effect is particularly significant, as it may lower the incidence or enhance the management of diabetic nephropathy, given the higher risk associated with poor glycemic control (Fioretto et al., 2006).

Few enteroendocrine hormones appear to have a negative effect on kidney disease, such as Neurotensin, Leptin and serotonin. *In vivo* data in mice demonstrated that high levels of serotonin may be associated with a poor prognosis in kidney disease (Takahashi et al., 2002; Kobayashi et al., 2008; Agarwal, et al., 2017). Therefore, the hypothesis that blockade of the serotonin receptor would be a good option in renal therapy is valid. However, a clinical trial carried out with sarpogrelate, a Serotonin receptor antagonist, at a dose of 300 mg/day for 4 weeks, did not demonstrate a renoprotective effect against acute kidney injury caused by contrast. Whether this blocker is beneficial in other kidney disease conditions remains to be investigated further.

## 5 Conclusion

**FIGURE 3:**
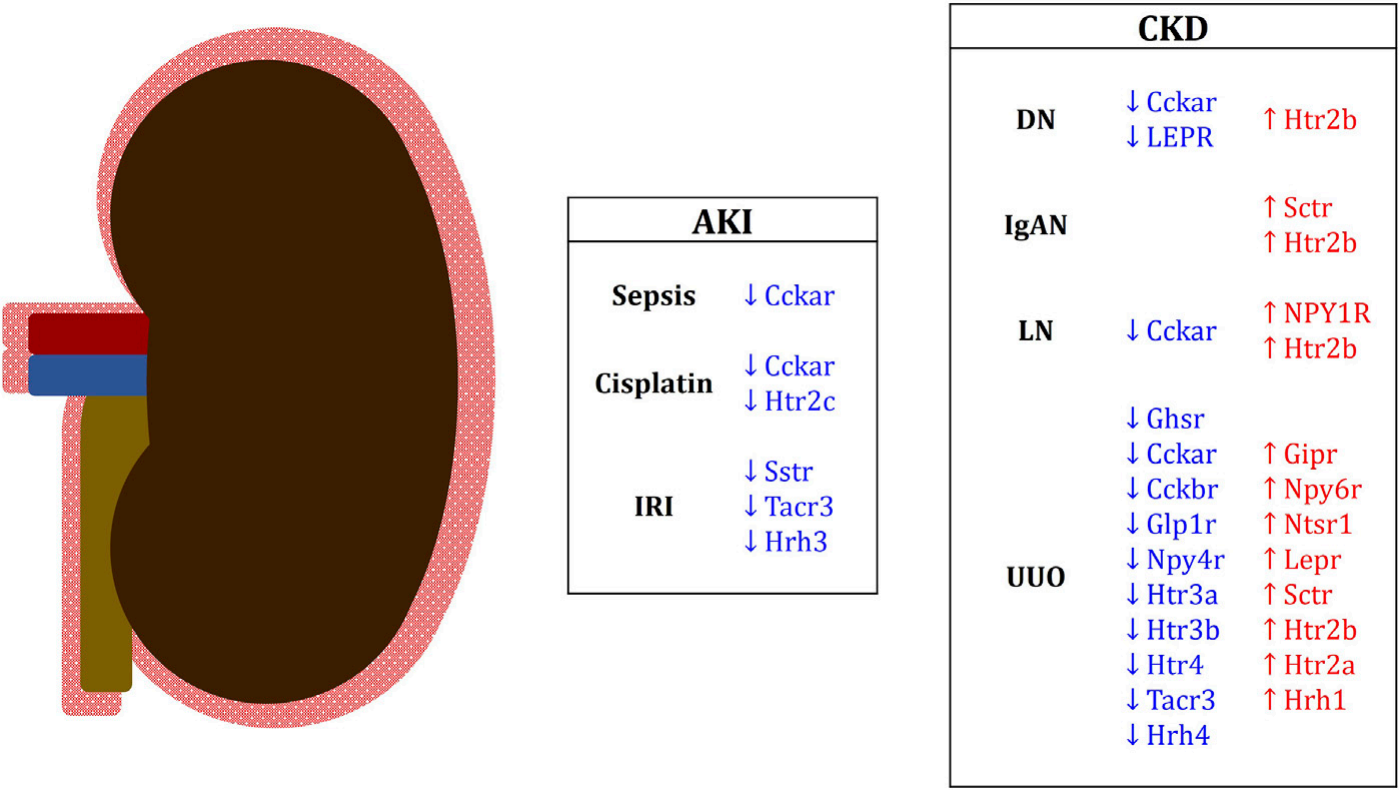
Differentially expressed EEC hormone receptors in renal conditions in mice and humans. Downregulated receptor genes are in blue; upregulated receptor genes are in red. Genes in capital letters are representative of human studies. AKI, acute kidney injury; IRI, ischemia-reperfusion injury; CKD, chronic kidney disease; DN, diabetic nephropathy; IgAN, IgA nephropathy; LN, lupus nephritis; UUO, unilateral ureteral obstruction.

Collectively, EECs serve as the source of a wide range of hormones capable of exerting extraintestinal effects. In general, with few exceptions, EEC hormones exhibit renoprotective properties due to their anti-inflammatory effects on the kidneys. The main mechanisms involve the inhibition of intracellular signaling pathways associated with pro-inflammatory and pro-fibrotic factors, such as NF-κB and TGF-β, respectively, in glomerular, tubular cells, and resident immune cells. These effects on renal tissues may be further reinforced by the presence of various EEC hormone receptors expressed within the renal parenchyma. Since each EEC hormone receptors can be selectively stimulated by specific hormones, and the distribution of these receptors may vary within the renal parenchyma, it is likely that the role of EEC hormone receptors stimulation contributes to the pro-inflammatory and pro-fibrotics signaling pathways observed in different types of kidney diseases with context-dependent effects. Indeed, the *in silico* data presented here demonstrates that some of these receptors can be differentially expressed depending on the evaluated kidney disorders (Figure 3), which reinforces the importance of the role of EEC hormones in kidneys and open new fields for mechanism investigation and therapeutic interventions. As described in Table 2, randomized clinical trials are exploring the therapeutic potential of EEC hormones in kidney disease. However, additional studies are imperative to delve into the specific role of these hormones within kidney cells, particularly in the context of renal diseases. It is essential to ascertain whether renal diseases have the potential to disrupt the biology of EECs, thereby influencing the production of EEC hormones. Such investigations may shed light on the existence of a positive feedback mechanism, potentially exacerbating kidney dysfunctions.
